# Leveraging electronic medical records to evaluate a computerized decision support system for staphylococcus bacteremia

**DOI:** 10.1038/s41746-025-01569-3

**Published:** 2025-03-28

**Authors:** Julia Palm, Ssuhir Alaid, Danny Ammon, Julian Brandes, Andreas Dürschmid, Claudia Fischer, Jonas Fortmann, Kristin Friebel, Sarah Geihs, Anne-Kathrin Hartig, Donghui He, Andrew J. Heidel, Petra Hetfeld, Roland Ihle, Suzanne Kahle, Verena Koi, Margarethe Konik, Frauke Kretzschmann, Henner Kruse, Norman Lippmann, Christoph Lübbert, Gernot Marx, Rafael Mikolajczyk, Anne Mlocek, Stefan Moritz, Christoph Müller, Susanne Müller, Ariadna Pérez Garriga, Lo An Phan-Vogtmann, Diana Pietzner, Mathias W. Pletz, Mario Popp, Maike Rebenstorff, Jonas Renz, Florian Rißner, Rainer Röhrig, Kutaiba Saleh, Sebastian G. Schönherr, Cord Spreckelsen, Anja Stempel, Abel Stolz, Eric Thomas, Susanne Thon, Daniel Tiller, Sebastian Uschmann, Sebastian Wendt, Thomas Wendt, Philipp Winnekens, Oliver Witzke, Stefan Hagel, André Scherag

**Affiliations:** 1 Institute of Medical Statistics, Computer and Data Sciences, Jena University Hospital, Jena, Germany; 2 IT Department, Data Integration Center, University Hospital Halle, Halle, Germany; 3 Data Integration Center, Jena University Hospital, Jena, Germany; 4 Center for Medical Informatics, Data Integration Center, University of Leipzig Medical Center, Leipzig, Germany; 5 Institute of Medical Informatics, University Hospital RWTH Aachen, Aachen, Germany; 6 IT Department, Data Integration Center, University Hospital Aachen, Aachen, Germany; 7 Central IT Department, Data Integration Center, University Hospital Essen, Essen, Germany; 8 Department of Intensive Care Medicine and Intermediate Care, University Hospital RWTH Aachen, Aachen, Germany; 9 Department of Infectious Diseases, West German Centre of Infectious Diseases, University Hospital Essen, University Duisburg-Essen, Essen, Germany; 10 Institute for Medical Microbiology and Virology, University Hospital Leipzig, Leipzig, Germany; 11 Division of Infectious Diseases and Tropical Medicine, Department of Medicine I, University Hospital Leipzig, Leipzig, Germany; 12 Institute of Medical Epidemiology, Biometrics, and Informatics, Medical Faculty of the Martin-Luther University Halle-Wittenberg, Halle, Germany; 13 Section of Clinical Infectious Diseases, University Hospital Halle, Halle, Germany; 14 Institute for Infectious Diseases and Infection Control, Jena University Hospital, Friedrich-Schiller-University Jena, Jena, Germany; 15 Center for Clinical Studies, Friedrich-Schiller-University Jena, Jena, Germany; 16 Institute of Medical Microbiology, Jena University Hospital, Jena, Germany; 17 Hospital Hygiene Staff Unit, University Hospital Halle (Saale), Halle, Germany

**Keywords:** Bacterial infection, Medical research

## Abstract

Infectious disease specialists (IDS) improve outcomes of patients with Staphylococcus bacteremia, but immediate IDS access is not always guaranteed. We investigated whether a care-integrated computerized decision support system (CDSS) can safely enhance the standard of care (SOC) for these patients. We conducted a multicenter, noninferiority, interventional stepped-wedge cluster randomized controlled trial relying on the data integration centers at five university hospitals. By this means, electronic medical records can be used for part of the trial documentation. We analyzed 5056 patients from 134 wards (*Staphylococcus* *aureus* (SAB): *n* = 812, coagulase-negative staphylococci (CoNS): *n* = 4244) and found that the CDSS was noninferior to the SOC for hospital mortality in all patients. Noninferiority regarding the 90-day mortality/relapse in SAB patients was not observed and there was no evidence for differences in vancomycin usage among CoNS patients. Despite low reported usage, physicians rated the CDSS’s usability favorably. **Trial registration**: drks.de; Identifier: DRKS00014320; Registration Date: 2019-05-06.

## Introduction

Staphylococci, namely, *Staphylococcus aureus* and coagulase-negative staphylococci (CoNS), play a major role in bloodstream infections, and *S. aureus* bacteremia (SAB) is associated with a high mortality risk and frequent recurrence^1^. The management of SAB relies on adherence to treatment recommendations, including the judicious selection of antibiotics^2–6^. Conversely, CoNS bloodstream infections pose a clinical challenge, as many episodes involve mere contaminants, leading to unnecessary antibiotic use, subsequent adverse events, antibiotic resistance, and increased costs^7,8^. Optimal management for staphylococcal bloodstream infections must meet two essential criteria: adherence to recommended treatment principles and prompt administration. Ideally, the immediate involvement of an infectious disease specialist (IDS) ensures prompt and optimal treatment for every patient with a staphylococcal bloodstream infection^5,9–11^. However, these specialists are scarce in many parts of the world. One solution to mitigate this scarcity is the adoption of algorithm-based therapy, as explored by Holland et al.^12^. Their study demonstrated that algorithm-determined antibiotic treatment resulted in a noninferior clinical outcome compared to the established standard of care (SOC) but did not improve adverse events. Unsolicited IDS telephone consultations for nonacademic hospitals were suggested as a middle ground between comprehensive IDS service and pure algorithmic support. However, this approach did not lead to a reduction in 30-day all-cause mortality compared to SOC without consultation among SAB patients in a recent clinical trial^13^, which was attributed to the time delay between the onset of bacteremia and the IDS consultation. Based on these findings, we propose that combining a CDSS with IDS consultation could overcome the limitations of each approach. The CDSS could guide physicians until an IDS steps in by bridging the gap between infection detection and specialized consultation. In the HELP trial (**H**ospital-wide **El**ectronic computerized decision support system to improve outcomes of **P**atients), we investigated the use of a CDSS to assist physicians in implementing best practices for patients with staphylococcal bacteremia. The “HELP CDSS” aims to optimize IDS allocation and patient outcomes while also serving as a use case for the newly established Data Integration Centers (DIC)^14^ within the Medical Informatics Initiative Germany^15^. This initiative seeks to enable the integration of diverse clinical routine data for the enhancement of patient care and scientific applications. Given the logistical constraints of having to implement the CDSS across entire wards, the HELP trial was designed as a stepped-wedge cluster randomized trial.

## Results

### Patient and ward flow throughout the trial

**Fig. 1:**
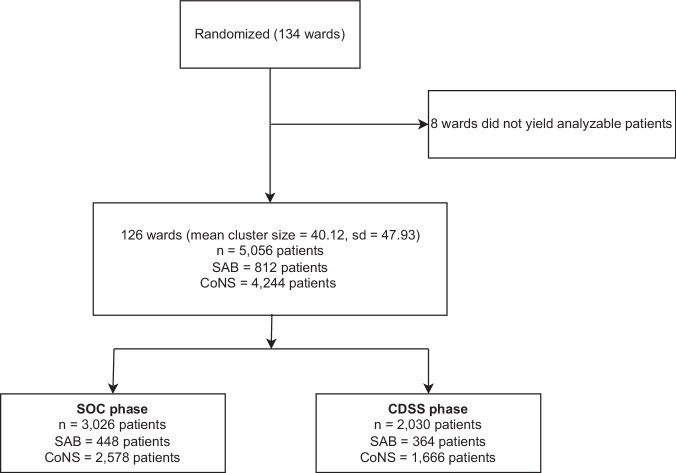
Recruitment CONSORT flowchart. Due to the stepped-wedge design, each ward took part in the SOC phase at the beginning of the trial and switched to the CDSS phase at a randomized crossover point in time, thus contributing patients from both phases to the analysis. Numbers of patients are displayed for each type of bacteremia separately.

In the five DIC, 5056 patients with Staphylococcus bacteremia were identified across 126 out of the 134 initially randomized wards. No patients were found in the remaining 8 wards during the study period. Among the identified patients, 812 were diagnosed with SAB, and 4244 were diagnosed with CoNS (Fig. 1). The baseline characteristics of the patients are detailed in Table 1, and SAB- and CoNS-specific data are provided in Supplementary Tables 2 and 3.

**Table 1:** Total sample characteristics

		Total (*n* = 5056)	SOC phase (*n* = 3026)	CDSS phase (*n* = 2030)
Demographics	Male	3450 (68.24%)	2066 (68.27%)	1384 (68.18%)
	Age (years)	64.34 [61.92; 66.77]	64.53 [61.77; 67.29]	64.19 [62.13; 66.26]
Hospital care	Length of stay (days)	32.1 [29.33; 34.87]	31.29 [27.6; 34.99]	33.15 [30.05; 36.26]
	Hemodialysis	1318 (26.07%)	823 (27.2%)	495 (24.38%)
	Days on dialysis	14.17 [3.14; 25.19]	14.34 [1.63; 27.05]	9.06 [6.77; 11.34]
	Mechanical ventilation	1861 (36.81%)	1263 (41.74%)	598 (29.46%)
	Ventilation days	16.75 [11.43; 22.07]	16.46 [10.67; 22.25]	17.15 [12.97; 21.33]
Bilirubin (µmol/l)	Baseline^a^	91.33 [−52.46; 235.11]	100.94 [−64.73; 266.61]	77.93 [−38.82; 194.69]
	Hospital discharge	117.03 [−58.78; 292.84]	119.53 [-64.69; 303.74]	105.06 [−49.26; 259.38]
	Difference (baseline-discharge)	−6.51 [−15.04; 2.01]	−4.45 [−12.51; 3.61]	−4.56 [−11.15; 2.02]
ASAT (µmol/l*s)	Baseline^a^	2.14 [1.29; 2.99]	2.03 [1.07; 3]	1.87 [1.18; 2.57]
	Hospital discharge	8.04 [2.7; 13.37]	8.79 [4.18; 13.41]	5.78 [−0.13; 11.7]
	Difference (baseline-discharge)	−5.1 [−8.88; −1.32]	−4.85 [−8.83; −0.87]	−3.17 [−7.41; 1.08]
ALAT (µmol/l*s)	Baseline^a^	1.28 [1.13; 1.44]	1.18 [0.95; 1.41]	1.43 [1.15; 1.7]
	Hospital discharge	3.13 [1.05; 5.21]	3.09 [1.42; 4.76]	2.5 [0.11; 4.89]
	Difference (baseline-discharge)	−1.68 [−3.49; 0.13]	−1.65 [−2.91; −0.39]	−0.99 [−3.68; 1.7]
Creatinine clearance (ml/min)	Baseline^a^	72.52 [48.84; 96.2]	71.23 [48.8; 93.67]	69.97 [47.85; 92.08]
	Hospital discharge	70.68 [53.62; 87.74]	70.37 [52.53; 88.21]	65.11 [48.33; 81.88]
	Difference (baseline-discharge)	−1.17 [−4.46; 2.12]	1.54 [-1.13; 4.2]	−4.18 [−7.98; −0.38]

Percent for binary variables mean [95% CI] for metric variables.

^a^Day of first staphylococcal blood culture.

### Patient-related outcomes

**Fig. 2:**
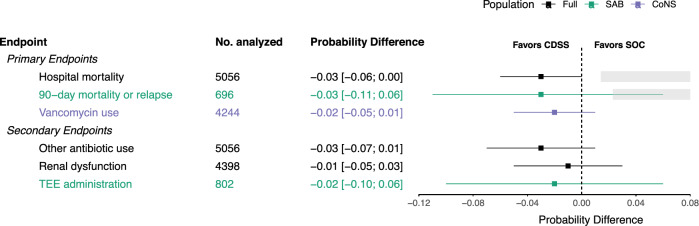
Differences in endpoint probability between the SOC and CDSS phases. The gray boxes show the area of noninferiority (=5% of the SOC phase probability). Overlapping CIs indicate that noninferiority could not be established, while the CIs left of the gray box indicate noninferiority. Confidence intervals: 90% for noninferiority hypotheses and 95% for superiority hypotheses.

Overall hospital mortality was 27% (1365 of 5056 patients), with 26% (797 of 3026 patients) during the SOC phase and 28% (568 of 2030 patients) during the CDSS phase. Among the SAB patients, the 90-day mortality was 32% (262 of 812 patients), with 31% (138 of 448 patients) and 34% (124 of 364 patients) during the SOC and CDSS phases, respectively. The 90-day relapse rate for SAB patients was 7% (54 of 812 patients), with 7% (33 of 448 patients) during the SOC phase and 6% (21 of 364 patients) during the CDSS phase. Figure 2 displays the GLMM-estimated probability differences of all outcomes and their confidence intervals.

The primary endpoint analysis revealed a hospital mortality of 28% in the SOC phase compared to 25% in the CDSS phase, indicating noninferiority of the CDSS phase (difference −3%; 90% CI −6% to 0%), given that +5% was not covered by the CI. With respect to the SAB patients, we next tested the subordinate primary endpoint of 90-day mortality or relapse, which was 46% in the SOC phase and 43% in the CDSS phase (difference -0.03; 90% CI −0.11 to 0.06), indicating that noninferiority was not met. Following the confirmatory test strategy, the subordinate primary endpoint limited to CoNS patients is reported only descriptively. Among patients with CoNS, 14% (351 of 2578 patients) were identified as having true CoNS infections in the SOC phase, as opposed to 17% (283 of 1666 patients) in the CDSS phase. We expected lower vancomycin use in the CDSS phase but found similar proportions in both phases: 26% in the SOC phase versus 24% in the CDSS phase (difference −2%; 95% CI −5% to 1%; *p* = 0.29). Additionally, the cumulative vancomycin dosage per patient did not significantly differ between phases: 10,298 mg in the SOC phase versus 12,251 mg in the CDSS phase (difference 1953 mg; 95% CI −120 mg to 4026 mg; *p* = 0.06). An overview of all the estimates is shown in Table 2. Figure 2 also summarizes the prespecified secondary endpoints. Although all three point estimates favored the CDSS, none of the estimates showed a significant effect. Sensitivity analyses, including COVID-19 diagnosis and site effects, yielded similar estimates, except for hospital mortality, where including site as a fixed effect changed the estimated difference in mortality (difference −4%; 90% CI −7% to −1%; *p* = 0.03). To investigate potential differences in hospital mortality between bacteremia types we conducted an exploratory post-hoc stratified analysis extending beyond our pre-registered plan. For SAB, crude mortality rates were 40% (129 out of 319 patients) in the SOC phase vs. 51% (123 out of 241 patients) in the CDSS phase. For CoNS patients, crude mortality rates were 35% (668 out of 1910 patients) in the SOC phase vs. 36% (445 out of 1221 patients) in the CDSS phase. Table 3 displays the results of the corresponding exploratory GLMM analysis.

**Table 2:** Model estimates for primary and secondary endpoints

		SOC	CDSS	Difference	*p*
Primary endpoints	Hospital mortality	0.28 [0.24; 0.32]	0.25 [0.21; 0.29]	−0.03 [−0.06; 0.00]^a^	0.09
	90-day mortality or relapse^b^	0.46 [0.38; 0.53]	0.43 [0.35; 0.51]	−0.03 [−0.11; 0.06]^a^	0.60
	Vancomycin use^c^	0.26 [0.22; 0.30]	0.24 [0.20; 0.28]	−0.02 [−0.05; 0.01]	0.29
	Cumulative Vancomycin use [mg]^c^	10298 [8693; 11902]	12251 [10216; 14286]	1953 [−120; 4026]	0.06
Secondary endpoints	Antibiotic use	0.40 [0.35; 0.44]	0.37 [0.32; 0.41]	−0.03 [−0.07; 0.01]	0.15
	Renal dysfunction	0.43 [0.39; 0.48]	0.42 [0.37; 0.47]	−0.01 [−0.05; 0.03]	0.65
	TEE administration^b^	0.75 [0.69; 0.81]	0.72 [0.66; 0.79]	−0.02 [−0.10; 0.06]	0.56

All estimates are probabilities, and all confidence intervals are 95% unless stated otherwise.

^a^90% CI for evaluating noninferiority.

^b^SAB patients only.

^c^CoNS patients only.

**Table 3:** Exploratory model estimates for hospital mortality stratified by type of bacteremia

	SOC	CDSS	Difference	*p*
SAB	0.35 [0.27; 0.42]	0.27 [0.21; 0.34]	0.07 [−0.01; 0.16]	0.09
CoNS	0.28 [0.24; 0.33]	0.23 [0.19; 0.27]	−0.05 [−0.09; −0.01]	0.02

Estimates represent probabilities and their 95% confidence intervals.

Additional site effect information can be found in Supplementary Table 5 and the crude mortality/relapse by site is available in Supplementary Tables 6 and 7.

### CDSS usage

**Fig. 3:**
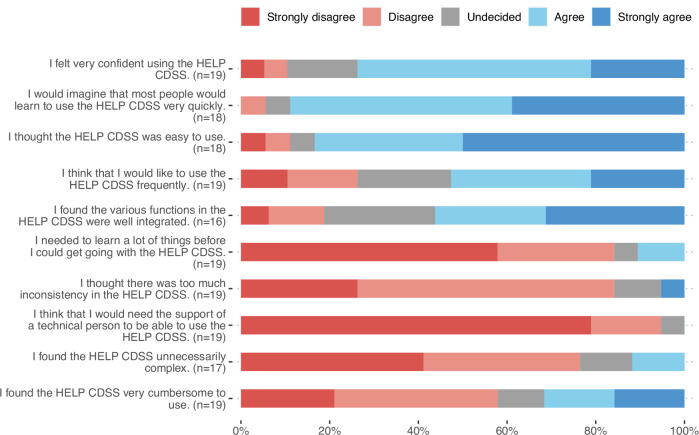
Usability rating of the HELP CDSS. Physicians rated 10 statements on the CDSS’s usability, using a scale from “strongly agree” to “strongly disagree.” Each bar shows the percentage of physicians who agreed with each category for each statement.

**Fig. 4:**
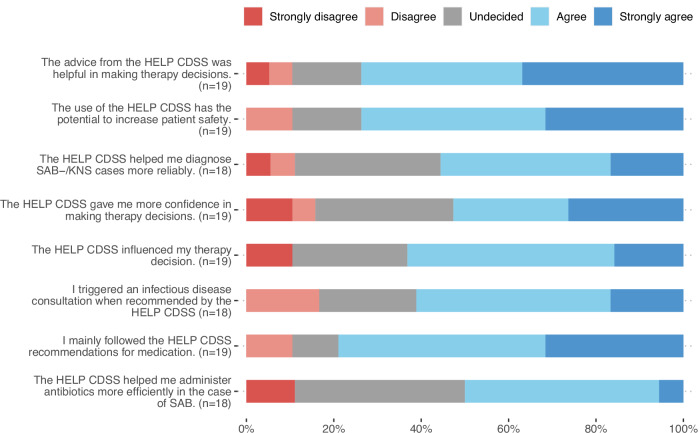
Impact of the CDSS on clinical decisions and patient safety. Physicians rated 10 statements on their clinical decisions and patient safety, using a scale from “strongly agree” to “strongly disagree.” Each bar shows the percentage of physicians who agreed with each category for each statement.

Forty physicians from all five hospitals participated in a poststudy survey to assess CDSS usage and satisfaction. Only 19 physicians used the CDSS, with 16 using it in just 25% of the bacteremia cases they encountered. The survey, which was conducted six months after this study, might have missed some CDSS users due to high staff turnover rates. Despite the low usage rate of the CDSS, Fig. 3 shows that the system had consistently high usability ratings, suggesting that the issue was not CDSS usability. Instead, free-text responses pointed to the limited accessibility of the CDSS due to suboptimal clinical IT service integration. Nevertheless, Table 4 (first line) indicates a 14% reduction in IDC during the CDSS phase versus the SOC phase, while most infection management parameters remained consistent, suggesting that the CDSS could uphold treatment adherence in these patients. This finding aligns with the clinicians’ subjective assessment (Fig. 4), which showed high compliance with the CDSS recommendations.

**Table 4:** Management of SAB infections

Management	Total (*n* = 812)	SOC phase (*n* = 448)	CDSS phase (*n* = 364)
Infectious disease consultation (IDC)	474 (58.37%)	289 (64.51%)	185 (50.82%)
Days until IDC	3.52 [−0.16; 7.21]	3.14 [0.04; 6.23]	3.51 [−0.42; 7.44]
At least one follow-up blood culture taken	685 (84.36%)	384 (85.71%)	301 (82.69%)
Number of follow-up blood cultures per case	0.83 [0.69; 0.97]	0.86 [0.75; 0.97]	0.81 [0.65; 0.97]
Days until follow-up blood culture taken	2.27 [0.6; 3.94]	1.97 [0.72; 3.22]	2.99 [−0.21; 6.18]
Removal of temp. vascular catheter	243 (29.93%)	141 (31.47%)	102 (28.02%)
Days until catheter removal	0.62 [−0.55; 1.79]	0.76 [0.05; 1.47]	0.7 [−1.82; 3.22]
Source control	257 (31.65%)	150 (33.48%)	107 (29.4%)
Duration until source control, days	2.34 [1.31; 3.37]	1.36 [0.47; 2.25]	3.28 [0.34; 6.22]
Oral sequential therapy	120 (14.78%)	66 (14.73%)	54 (14.84%)
Days of oral sequential therapy	28.11 [14.6; 41.62]	29 [13.84; 44.16]	25.6 [3.16; 48.04]
Combination therapy	235 (28.94%)	116 (25.89%)	119 (32.69%)
Days of combination therapy	8.39 [5.94; 10.84]	9.39 [5.8; 12.97]	6.99 [6.05; 7.92]

The square brackets indicate 95% confidence intervals.

## Discussion

The HELP trial evaluated the safety and benefits of CDSS support for treating Staphylococcus bacteremia. The CDSS phase was not inferior to the SOC phase in terms of hospital mortality. However, noninferiority for 90-day mortality/relapse in SAB patients could not be confirmed, and vancomycin use was not significantly reduced in CoNS patients. These results should be interpreted with caution, as the proportion of patients for whom the system was actually used can only be estimated. Originally, it was planned to monitor CDSS usage digitally, however this was not possible due to resistance from staff councils. Thus, we can only rely on the data from the poststudy survey, which suggest low usage. This can be attributed to various factors. First, the low self-reported usage of the CDSS may have stemmed from challenges in accessing the system, which is a common obstacle that has been identified in prior studies of CDSSs^16,17^. In anticipation of this challenge, we planned to integrate a HELP CDSS access point into all microbiology reports featuring Staphylococcus bacteremia, thus enabling immediate app launches with relevant patient data. However, this strategy was unfeasible due to IT security concerns. Second, all participating hospitals provided high-quality infectious disease management as part of the SOC, with dedicated infectious disease services that proactively approached relevant patients. Thus, matching and improving the already high SOC was difficult to achieve. The value of a CDSS might be greater in smaller hospitals without in-house IDSs, where it could bridge the gap between infection detection and telephone IDS consultation, as shown in the study by Weis et al.^13^. Although IDS consultation was theoretically mandatory for all SAB patients as part of the center’s standard of care (SOC), our data indicate that not all SAB patients actually received a consultation. This discrepancy can likely be attributed to two factors. First, some patients may have died before the consultation could occur. Second, the study was conducted during the COVID-19 pandemic, which has been reported to negatively impact the quality of care, particularly for SAB patients, as IDS were heavily engaged in managing COVID-19 cases^18^. Third, the COVID-19 pandemic caused major disruptions and limited the attention given to and thus usage of the HELP CDSS. Due to these challenges, the effect estimates of our primary analysis should be interpreted with caution. Nevertheless, the HELP trial demonstrated the potential of a CDSS to support Staphylococcus bacteremia treatment. Even in its technically simple form, physicians found the CDSS useful, suggesting a general appreciation for algorithmic decision support. The straightforward presentation of guideline information as an interactive decision tree may even be an advantage over more sophisticated yet less explainable artificial intelligence applications. In their recent editorial, Khera et al.^19^. caution against the hasty adoption of artificial intelligence-driven CDSSs, pointing out the potential risks of automation bias that could adversely affect doctors’ clinical decisions instead of enhancing them. The use of simpler and consequently more transparent systems, such as the HELP CDSS, may offer safer alternatives while still providing valuable assistance. This study has several limitations. First, the data collection process relied on post hoc identification of patients with Staphylococcus bacteremia through the integration of EHR data collected in the DIC. Because DIC infrastructures were still under development during the HELP trial, it is possible, albeit unlikely, that we missed patients in our analysis. Second, this study was conducted in academic hospitals with a high possibility of carry-over effects from physicians switching between wards in different phases. Finally, due to restrictions set by staff councils and the hospitals’ IT departments, we could not directly monitor CDSS usage for individual patients and therefore could not distinguish between intention-to-treat and per-protocol analyses. Overall, this stepped-wedge cluster randomized trial revealed that CDSS support for the treatment of Staphylococcus bacteremia was noninferior to SOC in terms of hospital mortality but did not significantly reduce vancomycin use. With its simple and MDR-compliant design, the CDSS received positive ratings from surveyed physicians, suggesting that it may improve patient outcomes if barriers to its use can be removed. Furthermore, the HELP study employed a hybrid data collection method that integrated the documentation of a traditional randomized controlled trial into clinical practice, contributing to the development of a learning healthcare system.

## Methods

### HELP computerized computerized decision support system

The HELP CDSS assigns patients to one of two arms: one for CoNS bacteremia (including *Staphylococcus intermedius*) or one for *S. aureus* bacteremia (including *Staphylococcus lugdunensis*). In the CoNS arm, the CDSS checks the number of independent blood cultures. If only one positive blood culture is available without evidence of catheter infection by the same pathogen (clinical judgment by the physician in charge), the CDSS recommends a follow-up blood culture to rule out contamination before starting antibiotics. If two separate blood cultures yield positive results for the same CoNS species with matching antibiotic susceptibility profiles, the CDSS suggests investigating a possible source of infection and considering antibiotic therapy. Blood cultures were considered separate if they were collected from different sites and at different times (2 h to 5 days). A negative follow-up blood culture suggested contamination, prompting a reevaluation of antibiotic therapy if it was already initiated.

**Fig. 5:**
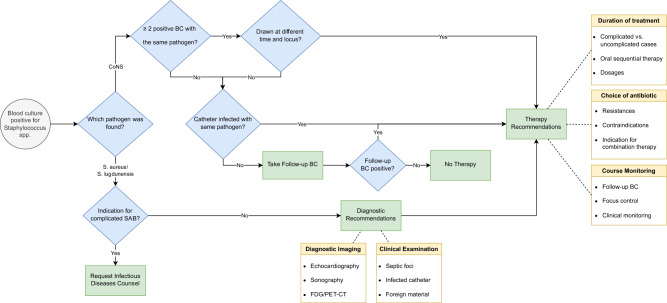
Schematic representation of the HELP CDSS. Rough overview of the decision algorithm that was the basis for the HELP CDSS.

The HELP CDSS for *S. aureus* bacteremia is based on treatment recommendations published by Hagel et al.^20^. It was developed by a panel of IDS and medical microbiologists in collaboration with medical informaticians from all study sites. A simplified schematic representation is shown in Fig. 5.

For SAB, the CDSS distinguishes between uncomplicated and complicated cases based on criteria such as positive follow-up blood cultures, permanent foreign bodies, prolonged fever, or vasopressor use. Treatment of complex SAB exceeds the capabilities of the CDSS, so immediate IDS consultation is advised. This also applies to polymicrobial bacteremia when more than one pathogen is detected in addition to staphylococci. For uncomplicated SAB treatment, guidance is offered, including source control, diagnostic imaging, and appropriate antibiotics.

Except for complicated SAB episodes, the CDSS can recommend treatment options independently of an IDS consultation. However, all hospitals maintained an IDS consultation service as part of their SOC, which remained operational throughout the treatment phase. Thus, the main difference between the two phases was that the CDSS provided supplementary information and acted as a bridge when immediate IDS consultation was not available. The CDSS was originally designed for seamless integration with hospital clinical information systems, but the development of this system faced challenges due to the European Medical Device Regulation (MDR)^21^, which was introduced in May 2021 and classified the original CDSS as a medical device requiring a highly complex and time-consuming certification process that was not compatible with the schedule. To ensure viability within our trial setting, the CDSS was transformed into a user input-reliant version that avoids automated patient-specific conclusions, functioning as an interactive decision tree accessible on smartphones, desktops, or laptops; an archived version of the CDSS is available online^22^.

### Study design

The HELP trial was designed as a stepped-wedge cluster randomized trial (SW-CRT)^23^ conducted at five German university hospitals: Jena, Leipzig, Aachen, Halle, and Essen. Following the methods outlined by Hussey and Hughes^24^^,^ we calculated a sample size of 135 wards enrolling at least 2700 patients based on a 90-day mortality of 30% among SAB patients, as reported by Mejer et al.^25^. For details, see the published trial protocol^26^. However, only 134 wards met the inclusion criteria described later and were included at randomization. All 134 wards started in the control phase (SOC) and transitioned to the treatment phase (HELP CDSS application) in a stepwise manner (Supplementary Fig. 1). The timing of crossover was determined by randomization, which was stratified by hospital and ward type (critical care units vs. general wards). Randomization lists generated using R^27^ were integrated into the technical infrastructure deploying the CDSS. This ensured that only physicians in the intervention wards could access the CDSS. There were nine randomization steps, with 2-month intervals for Jena, Leipzig, and Aachen and 1.5-month intervals for Halle and Essen due to legal difficulties in the pilot phase that necessitated a later start and thus a shorter overall data collection period. The trial adhered to Good Clinical Practice (where applicable) and the Declaration of Helsinki^28^, with approval from Jena University’s Ethics Committee (2018-1264_3-BO) and the respective center-specific ethics committees (Aachen University Ethics Committee, Halle University Ethics Committee, Essen University Ethics Committee, Leipzig University Ethics Committee.) The trial was registered at the German clinical trials register (www.drks.de/DRKS00014320) on June 6th 2019.

### Recruitment of wards and patients and CDSS rollout

We included all sufficiently technically equipped wards at each site, excluding maternity, psychiatry, and pediatric units. Physicians in intervention wards were informed about the impending CDSS rollout and received microbiology reports containing references to the CDSS, while wards in the control phase did not have CDSS access. We included all adult patients with a positive blood culture for *S. aureus*/*S. lugdunensis* or CoNS, except for CoNS patients who passed away within 72 h of the initial positive blood culture. Patients were included as new patients if they were discharged and then readmitted to the hospital or met the inclusion criteria more than 30 days after their initial inclusion. Study nurses monitored blood cultures and completed an electronic case report form (eCRF) for each HELP patient. Routine documentation collection did not require informed consent, except for SAB patients, who were scheduled for a 90-day telephone follow-up, with prior notification and the option to decline participation.

### Outcomes

The coprimary outcomes were hospital mortality for all patients, 90-day mortality/relapse for SAB patients and cumulative vancomycin use in milligrams for CoNS patients. Relapse was defined as the recurrence of *S. aureus* bacteremia or the occurrence of any related secondary complication within 90 days of the onset of initial SAB. We chose a pragmatic definition of relapse because without genotyping, it is difficult to ascertain whether a second infection is due to the same strain or infectious focus (i.e., relapse) or whether it is a reinfection with a different strain. Most *S. aureus* reinfections within 90 days are due to the same strain, suggesting the relapse of endogenous infection^29,30^. Secondary outcomes included the occurrence of acute renal dysfunction according to the Kidney Disease: Improving Global Outcomes (KDIGO)^31^ criteria, transesophageal echocardiography (TEE) usage and the use of seven additional antibiotics (Supplementary Table 1).

### Data collection

We implemented hybrid data collection, combining documentation in eCRFs with electronic health record (EHR) data collected in the five participating DIC. To enable secondary data use, DIC integrate EHR data from different clinical information systems and transform them into Fast Healthcare Interoperability Resources (HL7® FHIR®). This approach allowed the HELP trial to blend the characteristics of traditional randomized controlled trials using data from eCRFs with the practicality of being entirely embedded in the clinical routine. While this setup places restrictions on data availability and quality, it is much closer to a real-world clinical scenario. In addition, this approach has the potential to reduce the documentation burden associated with traditional trials, offering a more pragmatic study design within a learning healthcare system.

**Fig. 6:**
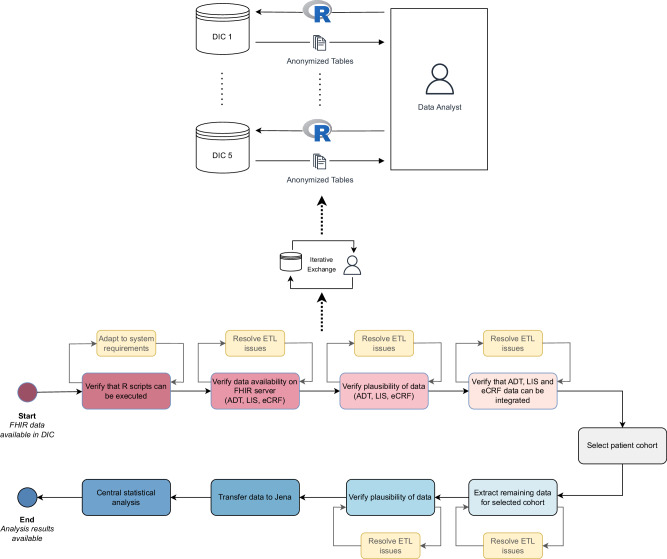
Data extraction process. Starting with FHIR data available in each DIC, each step of the data extraction required iterative adaptions until the local analysis result was ready to be sent to the central analysis site.

To identify patients with positive Staphylococcus blood cultures from participating wards in the DIC data, we sourced admission, discharge, and transfer (ADT) data and integrated them with data from the laboratory information system (LIS). Subsequently, all other data needed for downstream statistical analyses were extracted. To protect patient privacy, we used a distributed analysis approach in which R scripts using the fhircrackr^32^ package were sent to the DIC for preprocessing and local data aggregation, followed by central statistical analysis in Jena. Figure 6 shows the extraction process in more detail: we provided R code via GitLab to each of the five Data Integration Centers (DIC), tasked with extracting FHIR data from the FHIR server and converting it into tabular format. To ensure patient privacy, all identifying data were removed, and information relevant to clinical descriptions was aggregated. These anonymized data, depicted in the upper part of the figure, were subsequently sent to Jena. The creation of individual R scripts for each site necessitated extensive collaboration between the data analyst and the DIC employees, as outlined in the lower part of the figure. This process included verifying the executability of R scripts in the local IT environment, ensuring the availability and plausibility of FHIR data, and mapping relevant microbiology reports from the Laboratory Information System (LIS) to the associated electronic Case Report Form (eCRF) and admission, discharge, and transfer (ADT) data. The patient cohort was defined by selecting individuals with positive Staphylococcus blood cultures who were admitted to a HELP study ward when the first preliminary microbiology report was issued. Subsequently, data for this patient cohort were extracted from the FHIR server, checked for plausibility, and transmitted to Jena. Each of these steps required multiple iterations to ensure accuracy and functionality. After the study, we conducted an online survey incorporating the “System Usability Scale“^33^ among participating physicians to assess HELP CDSS usage and user satisfaction (see the Supplementary Materials).

### Statistical analysis

The coprimary outcomes were tested in a hierarchical, confirmatory fashion, meaning that each hypothesis test could only be interpreted as confirmatory if the null hypothesis for the preceding endpoint was rejected. For mortality outcomes, we used a noninferiority hypothesis with a 5% margin, indicating that our expectation was not to exceed a 5% increase in mortality in the CDSS vs. SOC phase. We employed binomial generalized linear mixed models (GLMMs) to estimate death/relapse probabilities and two-sided 90% confidence intervals (CIs). Regarding vancomycin use, our superiority hypothesis anticipated lower antibiotic usage in the CDSS phase. Extending the protocol descriptions, we used a two-part regression model. This included a binomial GLMM for vancomycin administration probability and a lognormal GLMM for cumulative dosage per patient. Analysis of the secondary outcomes followed the same modeling approach. In all the models, “treatment” (HELPS-CDSS vs. SOC) and “time since study initiation” were included as fixed effects; “ward” (cluster) was included as a random effect. To address potential site effects, we performed sensitivity analyses by adding “site” as a fixed effect in all the models and “coronavirus disease (COVID-19) diagnosis” as a fixed effect in all the mortality outcome models. Complying with privacy protection rules, we aggregated the variables used for the description of additional patient characteristics locally at each site and combined them using the meta-analytic random effects inverse variance model. All the statistical analyses were carried out with R version 4.2.1 using the R packages lme4^34^, marginaleffects^35^ and meta^36^.

## Supplementary information

Online supplementary materials

CONSORT Checklist

## Data Availability

Anonymized data collected for this study can be made available upon reasonable request and execution of appropriate data use agreements because health data are protected under Article 9(1) GDPR.

## Code availability

An archived version of the HTML code for the CDSS is available at 10.5281/zenodo.10704513. Access to the R code used for data analysis is available upon reasonable request from the corresponding author.

## References

[1] Diekema, D. J. et al. Survey of infections due to Staphylococcus species: frequency of occurrence and antimicrobial susceptibility of isolates collected in the United States, Canada, Latin America, Europe, and the Western Pacific Region for the SENTRY Antimicrobial Surveillance Program, 1997–1999. *Clin. Infect. Dis.* **32**, S114–S132 (2001). 11320452 10.1086/320184

[2] Schmitt, S. et al. Infectious diseases specialty intervention is associated with decreased mortality and lower healthcare costs. *Clin. Infect. Dis.* **58**, 22–28 (2014). 24072931 10.1093/cid/cit610

[3] Vogel, M. et al. Infectious disease consultation for *Staphylococcus aureus* bacteremia—a systematic review and meta-analysis. *J. Infect.* **72**, 19–28 (2016). 26453841 10.1016/j.jinf.2015.09.037

[4] Benfield, T. et al. Increasing incidence but decreasing in-hospital mortality of adult *Staphylococcus aureus* bacteraemia between 1981 and 2000. *Clin. Microbiol. Infect.* **13**, 257–263 (2007). 17391379 10.1111/j.1469-0691.2006.01589.x

[5] López-Cortés, L. E. et al. Impact of an evidence-based bundle intervention in the quality-of-care management and outcome of *Staphylococcus aureus* bacteremia. *Clin. Infect. Dis.* **57**, 1225–1233 (2013). 23929889 10.1093/cid/cit499

[6] Kern, W. V. Management of *Staphylococcus aureus* bacteremia and endocarditis: progresses and challenges. *Curr. Opin. Infect. Dis.* **23**, 346–358 (2010). 20592532 10.1097/QCO.0b013e32833bcc8a

[7] Weinstein, M. P. et al. The clinical significance of positive blood cultures in the 1990s: a prospective comprehensive evaluation of the microbiology, epidemiology, and outcome of bacteremia and fungemia in adults. *Clin. Infect. Dis.* **24**, 584–602 (1997). 9145732 10.1093/clind/24.4.584

[8] Souvenir, D. et al. Blood cultures positive for coagulase-negative staphylococci: antisepsis, pseudobacteremia, and therapy of patients. *J. Clin. Microbiol.* **36**, 1923–1926 (1998). 9650937 10.1128/jcm.36.7.1923-1926.1998 PMC104953

[9] Burnham, J. P. et al. Infectious diseases consultation reduces 30-day and 1-Year All-cause Mortality for Multidrug-resistant Organism Infections. *Open Forum Infect. Dis.* **5**, ofy026 (2018). 10.1093/ofid/ofy026 PMC5852998 29577058

[10] Jimenez-Aguilar, P., Lopez-Cortes, L. E. & Rodriguez-Bano, J. Impact of infectious diseases consultation on the outcome of patients with bacteraemia. *Ther. Adv. Infect. Dis.* **6**, 2049936119893576 (2019). 31839942 10.1177/2049936119893576 PMC6900613

[11] Madaline, T. et al. Early infectious disease consultation is associated with lower mortality in patients with severe sepsis or septic shock who complete the 3-hour sepsis treatment bundle. *Open Forum Infect. Dis.* **6**, ofz408 (2019). 31687417 10.1093/ofid/ofz408 PMC6821928

[12] Holland, T. L. et al. Effect of Algorithm-Based Therapy vs Usual Care on Clinical Success and Serious Adverse Events in Patients with Staphylococcal Bacteremia: A Randomized Clinical Trial. *JAMA* **320**, 1249–1258 (2018). 30264119 10.1001/jama.2018.13155 PMC6233609

[13] Weis, S. et al. Effect of automated telephone infectious disease consultations to nonacademic hospitals on 30-day mortality among patients with *Staphylococcus aureus* bacteremia: the SUPPORT Cluster Randomized Clinical Trial. *JAMA Netw. Open* **5**, e2218515 (2022). 35749114 10.1001/jamanetworkopen.2022.18515 PMC9233240

[14] Schreiweis, B., Ammon, D., Sedlmayr, M., Albashiti, F. & Wendt, T. in *gesundhyte.de : das Magazin für Digitale Gesundheit in Deutschland* Vol. 11 84–87 (2021).

[15] Semler, S. C., Wissing, F. & Heyder, R. German medical informatics initiative. *Methods Inf. Med.* **57**, e50–e56 (2018). 30016818 10.3414/ME18-03-0003 PMC6178199

[16] Catho, G. et al. Impact of interactive computerised decision support for hospital antibiotic use (COMPASS): an open-label, cluster-randomised trial in three Swiss hospitals. *Lancet Infect. Dis.* **22**, 1493–1502 (2022). 35870478 10.1016/S1473-3099(22)00308-5 PMC9491854

[17] Semler, M. W. et al. An electronic tool for the evaluation and treatment of sepsis in the ICU: a randomized controlled trial. *Crit. Care Med.* **43**, 1595–1602 (2015). 25867906 10.1097/CCM.0000000000001020 PMC4506222

[18] Arientova, S., Jicha, Z., Beran, O. & Holub, M. Decreased quality of care for *Staphylococcus aureus* bacteremia during the COVID-19 pandemic. *BMC Infect. Dis.* **22**, 631 (2022). 35854225 10.1186/s12879-022-07607-9 PMC9297622

[19] Khera, R., Simon, M. A. & Ross, J. S. Automation bias and assistive AI: risk of harm from AI-driven clinical decision support. *J. Am. Med. Assoc.* **330**, 2255–2257 (2023). 10.1001/jama.2023.22557 38112824

[20] Hagel, S., Weis, S. & Pletz, M. W. SOP management der *Staphylococcus aureus*—blutstrominfektion. *Intensivmed. up2date* **14**, 361–366 (2018).

[21] Regulation (EU) 2017/745 of the European Parliament and of the Council of 5 April 2017 on medical devices, amending Directive 2001/83/EC, Regulation (EC) No 178/2002 and Regulation (EC) No 1223/2009 and repealing Council Directives 90/385/EEC and 93/42/EEC 2017/05/05 2017. Legal Rule or Regulation. http://data.europa.eu/eli/reg/2017/745/oj.

[22] HELP CDSS v. V1.0 (SMITH - Smart Medical Information Technology for Healthcare, 2024).

[23] Hemming, K., Haines, T. P., Chilton, P. J., Girling, A. J. & Lilford, R. J. The stepped wedge cluster randomised trial: rationale, design, analysis, and reporting. *Br. Med. J.* **350**, h391 (2015). 25662947 10.1136/bmj.h391

[24] Hussey, M. A. & Hughes, J. P. Design and analysis of stepped wedge cluster randomized trials. *Contemp. Clin. Trials* **28**, 182–191 (2007). 16829207 10.1016/j.cct.2006.05.007

[25] Mejer, N. et al. Stable incidence and continued improvement in short term mortality of *Staphylococcus aureus* bacteraemia between 1995 and 2008. *BMC Infect. Dis.* **12**, 260 (2012). 23075215 10.1186/1471-2334-12-260 PMC3507819

[26] Hagel, S. et al. Hospital-wide ELectronic medical record evaluated computerised decision support system to improve outcomes of patients with staphylococcal bloodstream infection (HELP): study protocol for a multicentre stepped-wedge cluster randomised trial. *BMJ Open* **10**, e033391 (2020). 32047014 10.1136/bmjopen-2019-033391 PMC7044885

[27] R: A Language and Environment for Statistical Computing (R Foundation for Statistical Computing, Vienna, Austria, 2021).

[28] World Medical Association. Declaration of Helsinki: ethical principles for medical research involving human subjects. *J. Am. Med. Assoc.* **310**, 2191–2194 (2013). 10.1001/jama.2013.281053 24141714

[29] Fowler, V. G. Jr. et al. Recurrent *Staphylococcus aureus* bacteremia: pulsed-field gel electrophoresis findings in 29 patients. *J. Infect. Dis.* **179**, 1157–1161 (1999). 10191218 10.1086/314712

[30] Liao, C. H., Lai, C. C., Chen, S. Y., Huang, Y. T. & Hsueh, P. R. Strain relatedness of meticillin-resistant *Staphylococcus aureus* isolates recovered from patients with repeated bacteraemia. *Clin. Microbiol. Infect.* **16**, 463–469 (2010). 19614716 10.1111/j.1469-0691.2009.02885.x

[31] Mehta, R. L. et al. Acute Kidney Injury Network: report of an initiative to improve outcomes in acute kidney injury. *Crit. Care* **11**, R31 (2007). 17331245 10.1186/cc5713 PMC2206446

[32] Palm, J., Meineke, F. A., Przybilla, J. & Peschel, T. fhircrackr”: An R Package Unlocking Fast Healthcare Interoperability Resources for Statistical Analysis. *Appl. Clin. Inf.* **14**, 54–64 (2023). 10.1055/s-0042-1760436 PMC9876659 36696915

[33] Brooke, J. SUS: A quick and dirty usability scale. *Usability Eval. Ind.* **189**, 4–7 (1995).

[34] Bates, D., Mächler, M., Bolker, B. & Walker, S. Fitting linear mixed-effects models using lme4. *J. Stat. Softw.* **67**, 1–48 (2015).

[35] marginaleffects: Predictions, Comparisons, Slopes, Marginal Means, and Hypothesis Tests v. R package version 0.15.1 (2023).

[36] Balduzzi, S., Rucker, G. & Schwarzer, G. How to perform a meta-analysis with R: a practical tutorial. *Evid. Based Ment. Health* **22**, 153–160 (2019). 31563865 10.1136/ebmental-2019-300117 PMC10231495

