# Supplementary material for: Leveraging electronic medical records to evaluate a computerized decision support system for staphylococcus bacteremia
Source: NPJ Digit Med. 2025 Mar 28;8:180. doi: 10.1038/s41746-025-01569-3 (PMC11950190; doi:10.1038/s41746-025-01569-3)
Supplement: Supplementary file 1 — Online supplementary materials [file 41746_2025_1569_MOESM1_ESM.pdf]

Leveraging electronic medical records to evaluate a computerized decision support system for staphylococcus bacteremia

Supplementary Materials

Table Of Contents

Supplementary Table 1: Outcome variables in the HELP trial 2
Supplementary Table 2: CoNS sample characteristics....2
Supplementary Table 3: SAB sample characteristics....3
Supplementary Table 4: Comorbidities by ICD10 chapter 3
Supplementary Table 5: Model estimates from sensitivity analyses....4
Supplementary Table 6: Crude hospital mortality numbers stratified by site and type of bacteremia. 4
Supplementary Table 7: Crude 90-day mortality or relapse stratified by site 4
Supplementary Table 8: Number of wards randomized by site and ward type. 4
Supplementary Figure 1: Schematic representation of the stepped-wedge design....5
Detailed definitions and descriptions of the algorithm 6
Therapy recommendations: Substance Selection 7
Therapy recommendations: Therapy duration 8
Translation of the online usability survey 9

Supplementary Table 1: Outcome variables in the HELP trial.

Coprimary endpoints	Secondary endpoints	Technical and key performance indicators
• Hospital mortality<br>• Mortality or relapse within 90 days*<br>• Cumulative vancomycin use°	• Acute renal dysfunction (measured by creatinine)<br>• Administration of transesophageal echocardiographies*<br>• Use of: linezolid, daptomycin, vancomycin, flucloxacillin and cefazolin*<br>• Use of: linezolid, daptomycin, teicoplanin, fosfomycin, rifampicin°	• Number of ID consultations<br>• Adherence to HELP-CDSS recommendations<br>• Satisfaction with HELP-CDSS (by online survey after the study)

* SAB patients only, ° CoNS patients only

Supplementary Table 2: CoNS sample characteristics

		Total (n=4244)	SOC phase (n=2578)	CDSS phase (n=1666)
Type of infection	True CoNS infection	634 (14.94%)	351 (13.62%)	283 (16.99%)
	Health care associated	108 (2.54%)	48 (1.86%)	60 (3.6%)
	Nosocomial	578 (13.62%)	262 (10.16%)	316 (18.97%)
	Outpatient	132 (3.11%)	47 (1.82%)	85 (5.1%)
	Unknown	3426 (80.73%)	2221 (86.15%)	1205 (72.33%)
Foreign bodies[1]	Any permanent foreign body	803 (18.92%)	397 (15.4%)	406 (24.37%)
	Intracardiac device	135 (3.18%)	61 (2.37%)	74 (4.44%)
	Vascular prosthesis	37 (0.87%)	20 (0.78%)	17 (1.02%)
	Total hip replacement	67 (1.58%)	30 (1.16%)	37 (2.22%)
	Total knee replacement	52 (1.23%)	34 (1.32%)	18 (1.08%)
	Other joint prosthesis	12 (0.28%)	6 (0.23%)	6 (0.36%)
	Heart valve	67 (1.58%)	38 (1.47%)	29 (1.74%)
	Implanted vascular catheter	198 (4.67%)	103 (4%)	95 (5.7%)
	Other	378 (8.91%)	195 (7.56%)	183 (10.98%)
Source of infection	Focus identifiable	510 (12.02%)	288 (11.17%)	222 (13.33%)
	Vascular catheter	408 (9.61%)	228 (8.84%)	180 (10.8%)
	Cardiovascular system	29 (0.68%)	18 (0.7%)	11 (0.66%)
	Endocarditis	20 (0.47%)	12 (0.47%)	8 (0.48%)
	Intracardiac device	9 (0.21%)	5 (0.19%)	4 (0.24%)
	Intrathoracic	20 (0.47%)	13 (0.5%)	7 (0.42%)
	Urogenital/renal	9 (0.21%)	4 (0.16%)	5 (0.3%)
	CNS	10 (0.24%)	5 (0.19%)	5 (0.3%)
	Bone/joint	21 (0.49%)	11 (0.43%)	10 (0.6%)
	Intraabdominal	11 (0.26%)	8 (0.31%)	3 (0.18%)
	Skin/soft tissue	190 (4.48%)	97 (3.76%)	93 (5.58%)
	Postoperative wound infection	10 (0.24%)	3 (0.12%)	7 (0.42%)

[1]all foreign bodies were counted, irrespective whether they were infected or not

Supplementary Table 3: SAB sample characteristics

		Total (n=812)	SOC phase (n=448)	CDSS phase (n=364)
Risk factors	Intravenous drug abuse	25 (3.08%)	18 (4.02%)	7 (1.92%)
	Chronic hemodialysis	68 (8.37%)	34 (7.59%)	34 (9.34%)
	Cardiac risk factors	282 (34.73%)	178 (39.73%)	104 (28.57%)
Source of infection	Focus identifiable	559 (68.84%)	316 (70.54%)	243 (6.76%)
	Vascular catheter	182 (22.41%)	112 (25%)	70 (19.23%)
	Cardiovascular system	66 (8.13%)	39 (8.71%)	27 (7.42%)
	Intracardiac device	29 (3.57%)	17 (3.79%)	12 (3.3%)
	Intrathoracic	142 (17.49%)	71 (15.85%)	71 (19.51%)
	Urogenital/renal	27 (3.33%)	16 (3.57%)	11 (3.02%)
	CNS	11 (1.35%)	8 (1.79%)	3 (0.82%)
	Bone/joint	85 (10.47%)	47 (10.49%)	38 (10.44%)
	Intraabdominal	11 (1.35%)	8 (1.79%)	3 (0.82%)
	Skin/soft tissue	332 (40.89%)	187 (41.74%)	145 (39.84%)
	Postoperative wound infection	13 (1.6%)	9 (2.01%)	4 (1.1%)
Severity	Sepsis (within 7 days)	358 (44.09%)	201 (44.87%)	157 (43.13%)
	Septic shock	196 (24.14%)	113 (25.22%)	83 (22.8%)
	Endocarditis	70 (8.62%)	38 (8.48%)	32 (8.79%)
	Septic metastases	62 (7.64%)	30 (6.7%)	32 (8.79%)
	Positive FBC	147 (18.1%)	75 (16.74%)	72 (19.78%)
	Fever > 72h after therapy start	125 (15.39%)	67 (14.96%)	58 (15.93%)
	90-day death associated with S. aureus	143 (17.61%)	74 (16.52%)	69 (18.96%)

Supplementary Table 4: Comorbidities by ICD10 chapter

ICD10 Chapter	Total (n=4244)	SOC phase (n=2578)	CDSS phase (n=1666)
I: Certain infectious and parasitic diseases	314 (6.27%)	197 (6.63%)	117 (5.74%)
II: Neoplasms	976 (19.48%)	568 (19.11%)	408 (20.03%)
III: Diseases of the blood and blood-forming organs and certain disorders involving the immune mechanism	41 (0.82%)	24 (0.81%)	17 (0.83%)
IV: Endocrine, nutritional and metabolic diseases	65 (1.3%)	31 (1.04%)	34 (1.67%)
V: Mental and behavioural disorders	10 (0.2%)	8 (0.27%)	2 (0.1%)
VI: Diseases of the nervous system	123 (2.46%)	66 (2.22%)	57 (2.8%)
VII: Diseases of the eye and adnexa	5 (0.1%)	3 (0.1%)	2 (0.1%)
VIII: Diseases of the ear and mastoid process	1210 (24.16%)	729 (24.53%)	481 (23.61%)
IX: Diseases of the circulatory system	1094 (21.84%)	660 (22.21%)	434 (21.31%)
X: Diseases of the respiratory system	366 (7.31%)	231 (7.77%)	135 (6.63%)
XI: Diseases of the digestive system	34 (0.68%)	20 (0.67%)	14 (0.69%)
XII: Diseases of the skin and subcutaneous tissue	139 (2.78%)	80 (2.69%)	59 (2.9%)
XIII: Diseases of the musculoskeletal system and connective tissue	154 (3.07%)	82 (2.76%)	72 (3.53%)
XIV: Diseases of the genitourinary system	4 (0.08%)	4 (0.13%)	0 (0%)
XV: Pregnancy, childbirth and the puerperium	7 (0.14%)	5 (0.17%)	2 (0.1%)
XVI: Certain conditions originating in the perinatal period	47 (0.94%)	31 (1.04%)	16 (0.79%)
XVII: Congenital malformations, deformations and chromosomal abnormalities	369 (7.37%)	194 (6.53%)	175 (8.59%)
XVIII: Symptoms, signs and abnormal clinical and laboratory findings, not elsewhere classified	46 (0.92%)	34 (1.14%)	12 (0.59%)
XIX: Injury, poisoning and certain other consequences of external causes	5 (0.1%)	5 (0.17%)	0 (0%)

Supplementary Table 5: Model estimates from sensitivity analyses accounting for Covid-19 diagnosis (mortality endpoints only) and site (all endpoints).

		SOC phase	CDSS phase	Difference	p-value
Primary Endpoints	Hospital mortality	0.28 [0.24; 0.32]	0.25 [0.21; 0.29]	-0.04 [-0.07; -0.01] [1]	0.05
	90-day mortality or relapse*	0.47 [0.39; 0.54]	0.43 [0.34; 0.51]	-0.04 [-0.13; 0.05] [1]	0.46
	Vancomycin use°	0.24 [0.20; 0.28]	0.26 [0.22; 0.30]	-0.02 [-0.06; 0.01]	0.25
	Cumulative Vancomycin use [mg]°	10088 [8477; 11699]	12705 [10327; 15084]	2618 [412; 4823]	0.02
Secondary Endpoints	Antibiotic Use	0.40 [0.36; 0.44]	0.37 [0.33; 0.41]	-0.03 [-0.07; 0.01]	0.12
	Renal Dysfunction	0.44 [0.40; 0.47]	0.42 [0.37; 0.46]	-0.02 [-0.06; 0.03]	0.47
	TEE Administration*	0.75 [0.69; 0.81]	0.72 [0.66; 0.79]	-0.02 [-0.11; 0.07]	0.62

All estimates are probabilities and all confidence intervals are 95% unless stated otherwise.
* SAB patients only, ° KNS Patients only, [1] 90% CI for evaluating non-inferiority

Supplementary Table 6: Crude hospital mortality numbers stratified by site and type of bacteremia.

	Site 1 (n=612)	Site 2 (n=2008)	Site 3 (n=504)	Site 4 (n=894)	Site 5 (n=1038)
SAB	22/95 (23%)	55/228 (24%)	26/77 (34%)	55/176 (31%)	94/236 (40%)
CoNS	118/517 (23%)	431/1780 (25%)	120/427 (28%)	224/718 (31%)	220/802 (27%)

Supplementary Table 7: Crude 90-day mortality or relapse in the SAB sample stratified by site.

		Site 1 (n=95)	Site 2 (n=228)	Site 3 (n=77)	Site 4 (n=176)	Site 5 (n=236)
Relapse	Yes	3 (3%)	17 (7%)	6 (8%)	17 (10%)	11 (5%)
	No	58 (61%)	179 (79%)	65 (84%)	113 (64%)	162 (69%)
	Unknown	34 (36%)	32 (14%)	6 (8%)	46 (26%)	63 (26%)
Death	Yes	19 (20%)	74 (32%)	30 (39%)	43 (24%)	96 (41%)
	No	61 (64%)	138 (61%)	40 (52%)	118 (67%)	89 (38%)
	Unknown	15 (16%)	16 (7%)	7 (9%)	15 (9%)	51 (22%)
Relapse or death	Yes	22 (23%)	90 (39%)	36 (47%)	60 (34%)	106 (45%)
	No	58 (61%)	122 (54%)	34 (44%)	94 (54%)	74 (31%)
	Unknown	15 (16%)	16 (7%)	7 (9%)	22 (12%)	56 (24%)

Supplementary Table 8: Number of wards randomized by site and ward type.

Type of ward	Site 1	Site 2	Site 3	Site 4	Site 5	Overall
ICU/IMC	10	7	7	6	6	36
Normal	14	21	20	24	19	98
Total	24	28	27	30	15	134

Supplementary Figure 1: Schematic representation of the stepped-wedge design.

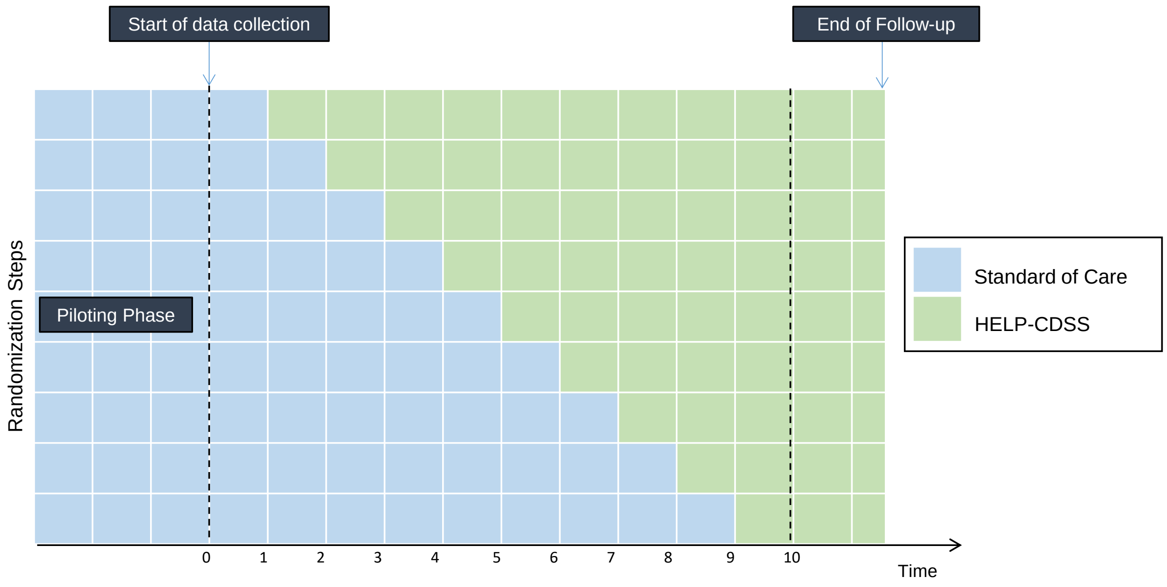

All wards began in the control phase (standard of care) and transitioned to the intervention phase (implementation of the HELP-CDSS) in a stepwise process. The nine predetermined crossover points were randomized to determine the timing of each ward’s switch.

Detailed definitions and descriptions of the algorithm

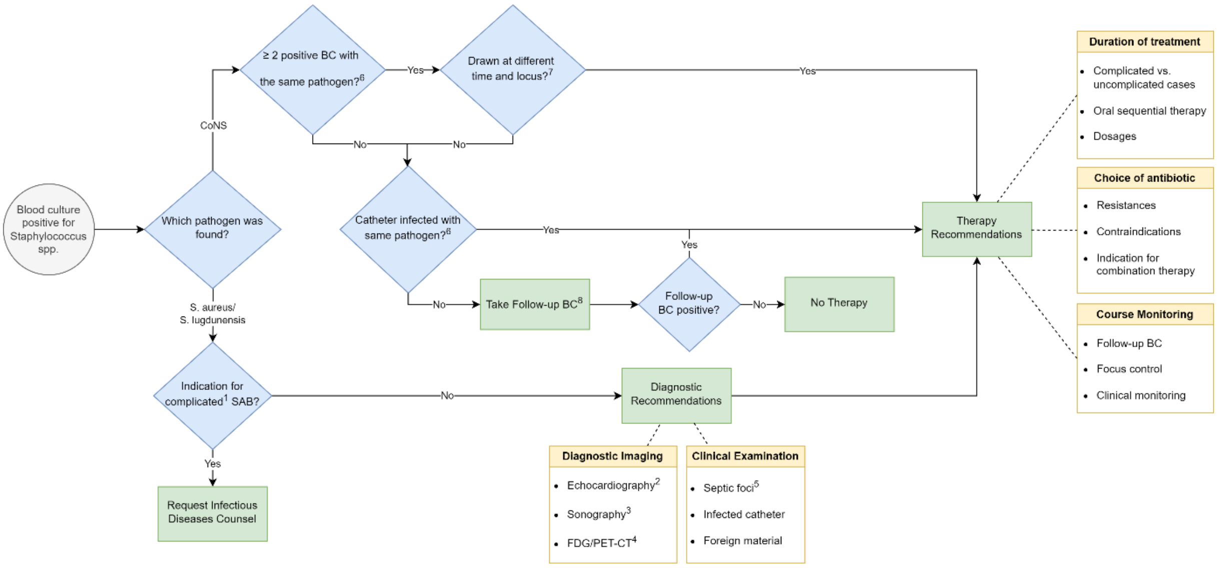

Footnotes:

1	Complicated SAB:<br>- Oxacillin resistance (MRSA)<br>- Need for vasopressors<br>- Intra- or extravascular foreign bodies<br>- Community-acquired bacteremia
2	Echocardiography indication:<br>- community-acquired bacteremia,<br>- cardiac risk factors present (cardiac foreign bodies, congenital heart disease, history of heart transplantation, history of endocarditis),<br>- septic emboli,<br>- vertebral osteomyelitis/osteomyelitis,<br>- hemodialysis,<br>- intravenous drug abuse
3	Sonography indication:<br>- suspicion of intraabdominal focus e.g. abscess,<br>- suspicion of pneumonia/empyema,<br>- suspicion of deep (postoperative) wound infection
4	FDG/PET-CT indication:<br>- positive blood cultures,<br>- unknown focus,<br>- clinical signs of septic dissemination (lung, head, abscess, etc.)
5	Septic Foci:<br>- Vertebral osteomyelitis : Tenderness to percussion?<br>- Joints : Swelling? Pain? Overheating?<br>- Endocarditis : Heart murmur? Skin signs? Janeway lesions, Osler's nodes, splinter hemorrhages, conjunctival bleeding?<br>- Wounds : (postoperative? chronic?) local signs of infection?<br>Pneumonia : Rales? Effusion? Empyema?
6	“Same pathogen” means species and suseceptibility testing are the same (i.e. no more than one deviation R/S/I)
7	BC should be drawn at least 2h apart but no more than 5 days.
8	Recommendation to take 2 pairs of BC again, one at the central catheter if available.

Therapy recommendations: Substance Selection

A) In the case of Oxacillin resistance

Backbone Therapy:

Vancomycin i.v.

	**Loading dose** 25-30 mg/kg body weight (use of actual body weight)<br>**Target level** 15–20 mg/l or with continuous infusion 20-25 mg/l<br>**Blood sampling for therapeutic drug monitoring** before next administration or before dialysis
**GFR >50**	**Maintenance dose** depending on trough level 15-20mg/kg every 8-12h or as a continuous infusion
**GFR 30-50**	
**GFR <30**	
**CHD**	1 g after dialysis or depending on concentration
**CVVH**	according to level

Or Daptomycin i.v.

	Note: Use of ideal body weight
**GFR >50**	1 x 10-12 mg/kg
**GFR 30-50**	1 x 8-10 mg/kg
**GFR <30**	1 x 8-10 mg/kg every 48h
**CHD**	1 x 8-10 mg/kg after dialysis
**CVVH**	1 x 8-10 mg/kg every 48h

If deep-seated focus or a severe disease or a non-infected (!) foreign body present:

Combination therapy with Rifampicin p.o. or i.v.

	Note: Use of ideal body weight
**GFR >50**	1 x 10 mg/kg distributed over 1-2 doses
**GFR 30-50**	
**GFR <30**	
**CHD**	
**CVVH**	

Or Combination therapy with Fosfomycin i.v.

	Note: Use of ideal body weight
**GFR >50**	3 x 5(-8*) g
**GFR 30-50**	3 x 3-5 g
**GFR <30**	2-3 x 2 g
**CHD**	4 g after dialysis
**CVVH**	3 x 5 g (up to 8 g)

*in case of meningitis and life-threatening infections

B) In the case of Oxacillin susceptibility

Backbone Therapy:

Flucloxacillin i.v.

**GFR >50**	6 x 2 g / 4 x 3 g
**GFR 30-50**	3 x 3 g
**GFR <30**	3 x 1-2 g
**CHD**	3 x 1 g
**CVVH**	3 x 4 g

Or Cefazolin i.v.

**GFR >50**	3-4 x 2 g
**GFR 30-50**	3 x 1 g
**GFR <30**	2 x 1 g
**CHD**	1 g after dialysis
**CVVH**	2 x 2 g

In case of penicillin allergy:

Daptomycin i.v.

	Note: Use of ideal body weight
**GFR >50**	1 x 10-12 mg/kg
**GFR 30-50**	1 x 8-10 mg/kg
**GFR <30**	1 x 8-10 mg/kg every 48h
**CHD**	1 x 8-10 mg/kg after dialysis
**CVVH**	1 x 8-10 mg/kg every 48h

Therapy recommendations: Therapy duration

A) In case of an uncomplicated infection (*exclusion of endocarditis, no implanted foreign bodies, no evidence of deep-seated metastatic deposits, none of the subsequent blood cultures were positive (for the same pathogen), fever reduction within 48-72h after starting therapy, infection focus known*)

-> Therapy duration 14 days i.v.

B) In case of a complicated infection

-> Determine therapy duration individually, usually at least 4-6 weeks.

Oral sequential therapy can be considered at the earliest after 14 days of intravenous antibiotic therapy. The prerequisite is that all of the following points are fulfilled:

- Adequate decrease of inflammation parameters
- Fever-free > 48h
- Source control
- Exclusion of endocarditis

C) Oral sequential therapy

<table>
<tr><td>Cefalexin</td><td><table><tr><td></td><td>Dosage: Cefalexin</td></tr><tr><td>**GFR >50**</td><td>3-4 x 1 g</td></tr><tr><td>**GFR 30-50**</td><td>3 x 0.5-1 g</td></tr><tr><td>**GFR <30**</td><td>2-3 x 500 mg</td></tr><tr><td>**CHD**</td><td>2x 0.5-1 g</td></tr><tr><td>**CVVH**</td><td>3-4 x 1 g</td></tr></table></td></tr>
<tr><td>**Or** Levofloxacin</td><td><table><tr><td></td><td>Dosage Levofloxacin p. o.</td></tr><tr><td>**GFR >50**</td><td>1-2 x 500 mg</td></tr><tr><td>**GFR 30-50**</td><td>1-2 x 250 mg</td></tr><tr><td>**GFR <30**</td><td>1-2 x 0.125 mg</td></tr><tr><td>**CHD**</td><td>1 x 0.125 mg</td></tr><tr><td>**CVVH**</td><td>1-2 x 500 mg</td></tr></table></td></tr>
<tr><td>**Or** Cotrimoxazole</td><td><table><tr><td></td><td>Dosage Cotrimoxazole p. o.</td></tr><tr><td>**GFR >50**</td><td>2-3 x 960 mg</td></tr><tr><td>**GFR 30-50**</td><td>2 x 960 mg</td></tr><tr><td>**GFR <30**</td><td>2 x 480 mg</td></tr><tr><td>**CHD**</td><td>1 x 480 mg</td></tr><tr><td>**CVVH**</td><td>3 x 960 mg</td></tr></table></td></tr>
<tr><td>**Or** Doxycycline</td><td><table><tr><td></td><td>Dosage Doxycycline p. o.</td></tr><tr><td>**GFR >50**</td><td rowspan="5">1 x 200 mg</td></tr><tr><td>**GFR 30-50**</td></tr><tr><td>**GFR <30**</td></tr><tr><td>**CHD**</td></tr><tr><td>**CVVH**</td></tr></table></td></tr>
<tr><td>**Or** Linezolid</td><td><table><tr><td></td><td>Linezolid dosage p. o.</td></tr><tr><td>**GFR >50**</td><td rowspan="5">2 x 600 mg</td></tr><tr><td>**GFR 30-50**</td></tr><tr><td>**GFR <30**</td></tr><tr><td>**CHD**</td></tr><tr><td>**CVVH**</td></tr></table></td></tr>
</table>

Translation of the online usability survey

1. At which location do you work?
   - o Aachen
   - o Essen
   - o Halle
   - o Jena
   - o Leipzig

2. What clinical professional experience do you have?
   - o 1 – 2 years as a resident physician
   - o More than 2 years as a resident physician
   - o Less than 10 years as a specialist physician
   - o More than 10 years as a specialist physician

   Personal specialty discipline and/or current field of deployment/clinic: [Free text box]

3. On which medium did you access the HELP CDSS?
   - o personal smartphone
   - o personal tablet
   - o work smartphone
   - o work tablet
   - o work laptop
   - o work desktop

4. How often do you use mobile IT (smartphone, laptop, tablet, etc.) in your clinical routine?
   - o multiple times daily
   - o daily
   - o once weekly
   - o never

5. How many times was the HELP CDSS consulted for decision-making upon request in the microbiological findings?
   I used the HELP CDSS:
   - o never
   - o in approximately 1 out of 4 cases
   - o in approximately 2 out of 4 cases
   - o in approximately 3 out of 4 cases
   - o in every case

6. Please provide a personal evaluation of the following statements using the 5-point scale provided. Mark the appropriate response for each statement; there are no right or wrong answers. Your personal conviction is all that matters.

Which statement do you think applies to the HELP CDSS?

	Completely disagree	Somewhat disagree	Undecided	Somewhat agree	Completely agree	Cannot judge
I found the HELP CDSS very cumbersome to use.						
I found the various functions in the HELP CDSS were well integrated.						
I think that I would like to use the HELP CDSS frequently.						
I found the HELP CDSS unnecessarily complex.						
I thought the HELP CDSS was easy to use.						
I think that I would need the support of a technical person to be able to use the HELP CDSS.						
I thought there was too much inconsistency in the HELP CDSS.						
I would imagine that most people would learn to use the HELP CDSS very quickly.						
I felt very confident using the HELP CDSS.						
I needed to learn a lot of things before I could get going with the HELP CDSS.						
The advice from the HELP CDSS was helpful in making therapy decisions.						
I triggered an infectious disease consultation when recommended by the HELP CDSS.						
I mainly followed the HELP CDSS recommendations for medication.						
The HELP CDSS influenced my therapy decision.						
The HELP CDSS gave me more confidence in making therapy decisions.						
The HELP CDSS helped me diagnose SAB-/KNS cases more reliably.						
The HELP CDSS helped me administer antibiotics more efficiently in the case of SAB.						
The use of the HELP CDSS has the potential to increase patient safety.						
I would like to continue using the HELP CDSS even after the study is completed.						
I would recommend the HELP CDSS to colleagues.						

7. How did you manage with the HELP CDSS?

   What did you like? [Free text box]

   Where there any problems? [Free text box]

   What could be improved about the HELP CDSS? [Free text box]
